# Structural Behavior of High-Strength Concrete Slabs Reinforced with GFRP Bars

**DOI:** 10.3390/polym13172997

**Published:** 2021-09-03

**Authors:** Maher A. Adam, Abeer M. Erfan, Fatma A. Habib, Taha A. El-Sayed

**Affiliations:** Department of Structural Engineering, Reinforced Concrete Structures, Shoubra Faculty of Engineering, Benha University, 108 Shoubra St., Shoubra, Cairo 11629, Egypt; maher.adam@feng.bu.edu.eg (M.A.A.); abeer.erfan@feng.bu.edu.eg (A.M.E.); Fatma.Habiba@feng.bu.edu.eg (F.A.H.)

**Keywords:** structural performance, exponential study, GFRP bars, high-strength concrete (HSC), nonlinear analysis, ANSYS 2019-R1

## Abstract

In this manuscript, structural testing was conducted on high-strength concrete slab specimens to investigate the behavior of such specimens when reinforced with a locally produced GFRP reinforcement. Subsequently, a finite element model (FEM) was constructed and validated against the experimental results. In the experimental phase, a total of eleven specimens (nine were reinforced with GFRP, while two were reinforced with conventional steel) were constructed and tested. The slabs dimensions are 700 mm × 1750 mm with variable thickness from 100 mm to 150 mm and different reinforcement ratios using different diameters. The structural behavior of the tested slabs was investigated in terms of ultimate load, ultimate deflection, load–deflection relationship, and crack pattern. Additionally, a nonlinear finite element model using the software ANSYS 2019-R1 was constructed to simulate the structural behavior of slabs reinforced with GFRP bars. The results obtained from the finite element analysis are compared with experimental results. The outcomes showed that the contribution of GFRP rebars in concrete slabs improved slab ductility and exhibited higher deflection when compared with traditional steel rebars. Good agreement between experimental and nonlinear analysis was obtained.

## 1. Introduction

Corrosion of steel reinforcing bars is one of the major problems that shorten the lifetime serviceability of reinforced concrete (RC) structures [1,2,3,4]. This has led to the development of new concrete-reinforcing materials. With their high strength and good corrosion resistance, fiber-reinforced polymers (FRPs) represent a good alternative. In comparison to steel, the distinctive properties of FRP materials are high strength, relatively low elastic modulus, and elastic response to failure. Given these different properties, the behavior of concrete elements reinforced with FRP is likely to differ markedly from those that employ conventional steel reinforcement. This difference is characterized not only by a different load–deflection response but also by a change in the mode of failure. The failure mechanism of FRP-reinforced concrete elements is due to them being relatively brittle, even in flexure. This gives rise to major concerns by structural engineers who are more familiar with the under-reinforced design philosophy developed for steel RC structures, which ensures a ductile failure to give plenty of warning. However, Ospina and Nanni [5] concluded that different deflections can be predicted for members reinforced with FRP bars that have similar stiffness but different ultimate tensile strength. Because deflection is a problem associated with the serviceability limit state, the procedure should not be linked to ultimate limit state parameters [6,7,8]. Lately, the flexural performance of FRP-RC elements has been widely studied. Benmokrane et al. [9] conducted an experimental and theoretical evaluation of the flexural performance of RC beams reinforced with glass FRP and steel. Masmoudi et al. [10] investigated the effects of reinforcement ratio on cracking patterns, deformation, flexural capabilities, and failure mechanisms of GFRP and steel-reinforced concrete beams. They evaluated the impact of compression reinforcement when calculating the final flexural capacity of the beams. Nonetheless, the impact was dismissed as insignificant. FRP and steel-reinforced concrete parts react differently in terms of serviceability. Alsayed et al. [11] found that GFRP beams could precisely anticipate flexural capabilities using the ultimate theory of designs. Toutanji and Saafi [12] changed the factor of the power in the equation of Branson to account for its experimental results with the elasticity of the bar and the ratio of reinforcement. Toutanji and Deng [13] demonstrated that ACI 440.1R-01 can successfully estimate deflections and crack width in one-layer FRP-bar beams with crack width. However, ACI 440.1R-01 may be employed once some parameters have been changed when FRP bars are arranged in two layers. Thiagarajan [14] reported the findings of an experimental and analytical investigation comparing the flexural performance of RC beams reinforced with sandblasted carbon basalt fiber rods composite rods. He studied 12 beams comprising three control steel beams that were evaluated for features of deformation and strength. Experimental results from pullout testing revealed that bonding of sandblasted rods is not a serious problem. The effective inertia prediction moment of the FRP-RC beam was investigated by Moussavi and Esfahani [15]. This article presents new equations based on evolutionary algorithms and experimental data to estimate the effective time of FRP-RC beams in inertia. The testing results were highly associated with the expected values using the suggested equations, particularly with high strengthening ratios and high load-levels. Rashid et al. [16] reported the flexural behavior of 10 HSC beams reinforced with aramid-fiber-reinforced polymers (AFRPs). The study recommended that ductility measurement for FRP beams is useful. The necessity to reduce the maximum distances between the stirrups as defined in the existing code has also been recognized, and recommendations have been given for sections with high shear forces coupled with considerable bending moments. Ashour [17] presented the flexural and shear capacity of 12 GFRP beams. Comparisons between the flexural capacity derived from theoretical analysis and those determined experimentally indicate satisfactory consent. Nayal and Rasheed [18] proposed a model investigating the tension stiffening of RC beams reinforced with steel and FRP bars. The study’s findings give useful model parameters for steel and FRP-RC beams. According to Kara and Ashour [19,20,21], the low elastic modulus of FRP bar results in significant crack width and deflections when compared with steel bars. Kassem et al. [22] investigated the serviceability of FRP beams reinforced with various types and reinforcement ratios. To assess the accuracy of such prediction models according to ACI Committee 440-H., the experimental results were compared with CSA 2002 and ACI Committee 440 2006 accessible models. Al-Sunna et al. [23] showed high ultimate capacities of moment compared obtained from nearly all codes. Barris et al. [24] evaluated the deflections and cracking in 14 GFRP RC beams for typical predicted models. The impact of the important factors was examined, and the appropriateness of various predicted models and the empirical coefficient modification were explored. Mahroug et al. [25,26] examined continuous concrete slabs strengthened with basalt and carbon FRP bars. The combined flexure–shear collapse mechanism was seen in all slabs. Furthermore, they demonstrated that increasing the bottom reinforcement of slabs is more successful than increasing the top reinforcement in enhancing load-carrying capacity and reducing midspan deflections. Dundar et al. [27] reported the load–displacement conduct of FRP and steel multispan RC beams. The deflection of FRP or steel RC beams was determined using a numerical technique. This study can offer a helpful method for calculating deflection for any type of reinforcement. Wang et al. [28] assessed the polymer tendons under sea conditions in both the prestressed basalt and hybrid fiber-reinforced tendons. The interior corrosive steel wires caused basalt and steel wire to degrade considerably more quickly. Chenggao et al. [29] investigated the distribution in a pultruded glass or carbon hybrid bar and absorption of water under temperatures and hydraulic pressures. The increased temperatures and hydraulic pressure accelerated the water diffusion in the hybrid bar. Demakos et al. [30] provided a numerical and experimental study for a structured curved frame. The thin-arched ultimate load achieved values similar to those seen for the mortar compressive strength employed. There was good agreement between experimental and numerical results. In an optimal design of a steel building, Papavasileiou and Pnevmatikos [31] submitted an investigation against an earthquake and the gradual cord collapse. This study indicates that the gradual collapse can bring the whole structure to failure locally by a structural component. The findings of this research show the promising cable potential as a way of increasing the building’s progressive resistance to collapse.

This paper presents the flexural behavior of one-way concrete slabs reinforced with locally manufactured GFRP bars. Currently, experimental data conducted for HSC slabs reinforced with GFRP bars are scarce. So, an experimental study was done to study the behavior of HSC slabs reinforced with GFRP bars with different reinforcement ratios varying from 0.8 µ_b_ to 1.2 µ_b_ (balanced reinforcement ratio) using different bars diameters. Eleven slabs 1750 mm in length, 700 mm in width, and 100 mm to 150 mm in depth were loaded and tested until failure. Nonlinear finite element analysis was conducted using ANSYS 2019-R1 to verify the obtained experimental results in terms of load–deflection curves, deflection, and crack pattern for all tested slabs.

## 2. Experimental Program

The experimental study was investigated in the Housing and Building National Research Center (HBNRC), Giza, Egypt. This study was performed to study the structural performance of HSC slabs reinforced with GFRP bars under flexural load. The ultimate load, ultimate deflection, concrete and GFRP bar strains, and crack pattern was obtained.

### 2.1. Experimental Study

#### 2.1.1. Concrete Mix

**Figure 1:**
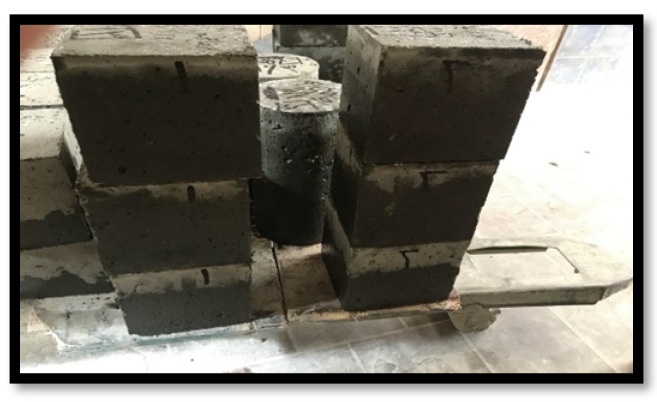
Concrete cubes.

The concrete mix of 60 MPa at 28 days compressive strength was used. Table 1 shows the weights of materials used. Concrete cubes were poured during pouring of the concrete slabs, as shown in Figure 1.

#### 2.1.2. Compressive Strength Test

**Figure 2:**
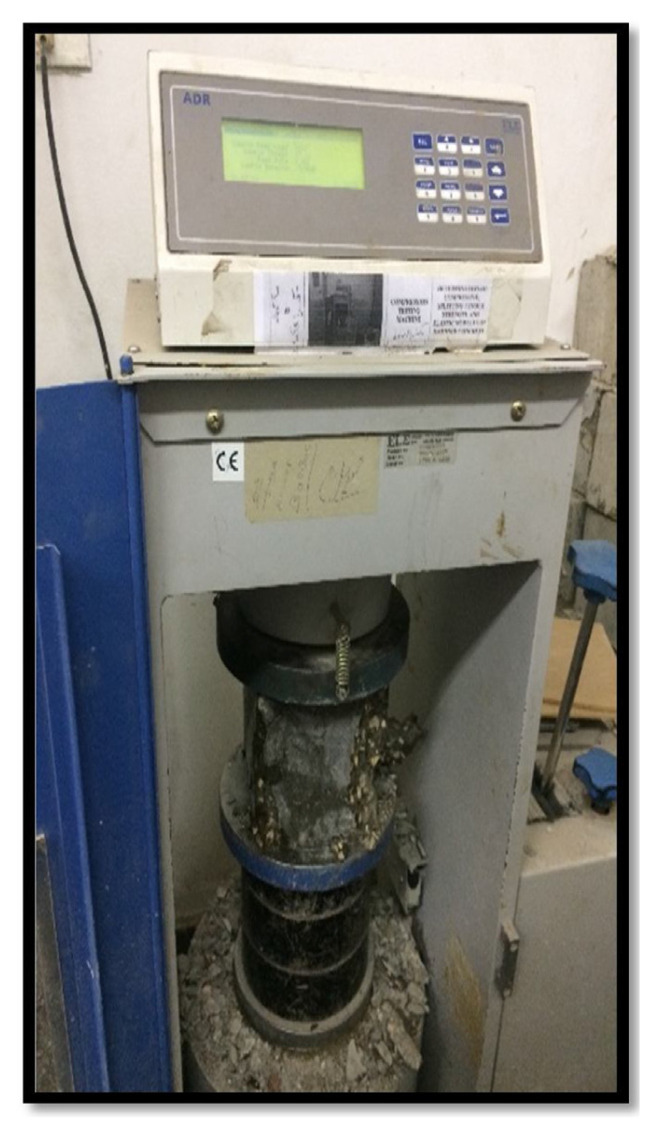
Concrete cubes under testing machine.

Concrete cubes of 150 × 150 × 150 mm dimensions were tested after 28 days under a universal testing machine of 2000 kN capacity for compression, according to ECP’2018 [32], as shown in Figure 2. Table 2 shows the compressive strength of the tested cubes.

#### 2.1.3. GFRP Bars

**Figure 3:**
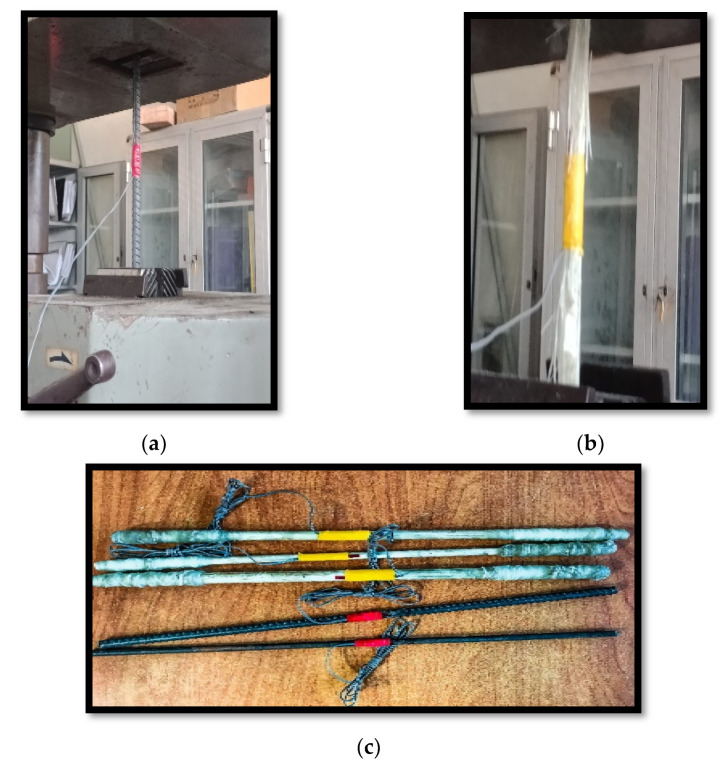
Tensile test: (**a**) steel bar; (**b**) GFRP bar of Φ 10 mm; (**c**) different bar diameters of Φ 8, 10, and 12 mm.

The tensile strength of used GFRP bars varied between 490, 650, and 750 MPa for diameters of 8 mm, 10 mm and 12 mm, respectively, as shown in Table 3. This tensile strength for nominal diameters of 8 mm, 10 mm, and 12 mm was tested in the Housing and Building National Research Center (HBNRC), as shown in Figure 3, according to ECP’2018 [32].

#### 2.1.4. Description of Tested Slabs

**Figure 4:**
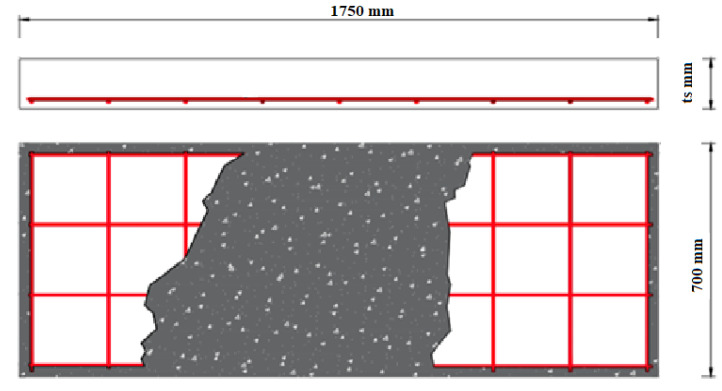
Slab dimensions and reinforcement.

The experimental program consists of four groups of concrete slabs with dimensions of 1750 mm in length and 700 mm in width and different heights from 100 mm to 150 mm. All tested slabs have the same 60 MPa compressive strength. The first group (SP1 and SP2) represents control slabs with balanced steel reinforcement ratios of 0.16 and 0.24, respectively, and concrete height of 100 mm. The second group is “Group I” (SP3, SP4, and SP5), with balanced fiber reinforcement ratios of 0.80, 1.00, and 1.20, respectively, and concrete height of 100 mm. The third group is “Group II” (SP6, SP7, and SP8), with balanced fiber reinforcement ratios of 1.20 and concrete height of 120 mm. The final group is “Group III” (SP9, SP10, and SP11), with balanced fiber reinforcement ratios of 1.20 and concrete height of 150 mm. Table 4 and Figure 4 showed the details for the tested slabs.

### 2.2. Test Setup

**Figure 5:**
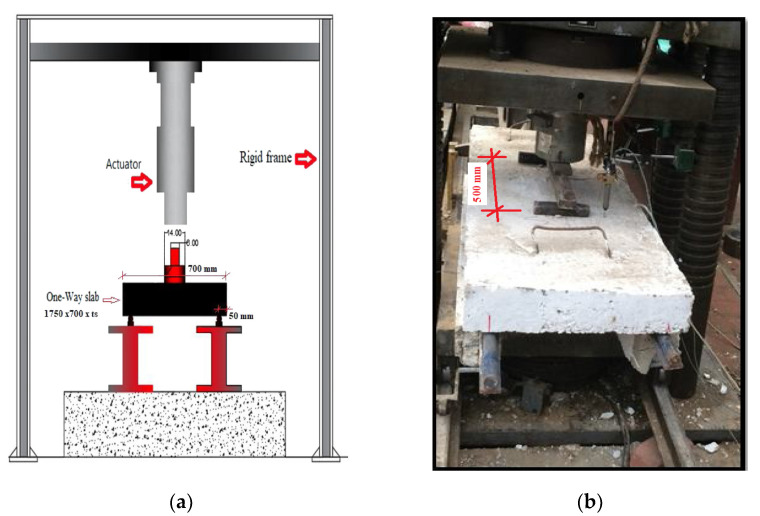

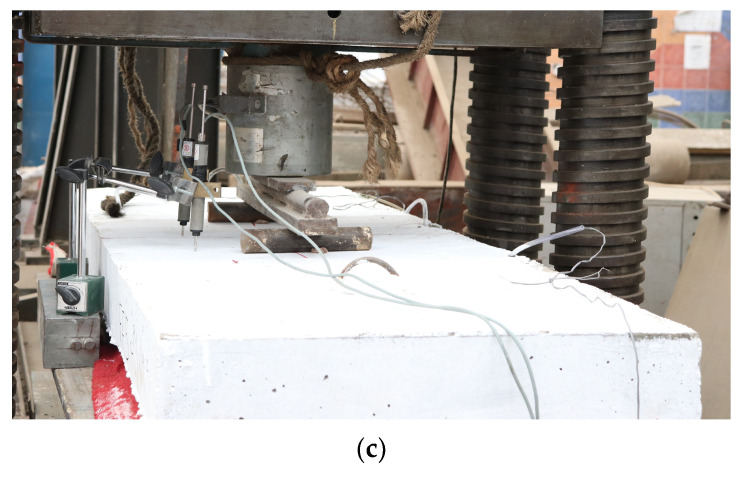
Test setup: (**a**) slab details, (**b**) flexural test setup, (**c**) LVDT and strain gauge locations.

Eleven HSC slabs were examined under two-point load with a 500 mm load distance, as in Figure 5. The test was performed in the National Building Research Center under a universal testing machine with a maximum capacity of 5000 KN. Outputs were recorded using LVDTs and strain gauges.

## 3. Experimental Results and Discussion

The results obtained from the experimental test were given in terms of ultimate load, ultimate deflection, load–deflection curves, crack pattern, and load strains for concrete and reinforcement rebars as follows.

### 3.1. Ultimate Load

Table 5 shows the ultimate load for all slabs. The ultimate load for the control group (SP1 and SP2) was 148.00 kN and 139.00 kN, respectively. This is due to the decreased diameter of bars and increased bonding between the concrete and steel bars, which agrees with the results recorded by Janus et al. [33].

For Group I (SP3, SP4, and SP5), Slab SP3 recorded the lowest ultimate load of 87.85 kN, which was also lower than the control slabs by a decreasing ratio of 39.0%. This is due to the small reinforcement ratio, which led to rupture of GFRP bars. However, for SP4 and SP5, the ultimate loads were 149.30 kN and 154.40 kN, respectively.

For Group II (SP6, SP7, and SP8), Slab SP6 recorded the highest ultimate load of 180.70 kN, which was higher than Slabs SP7 and SP8, in which the ultimate loads were 149.30 kN and 154.40 kN, respectively. It was recorded that a smaller diameter indicated high performance with concrete slabs as in SP6, which recorded an ultimate load of 180.70 kN, higher than that obtained from Slabs SP7 and SP8, which recorded 149.30 kN and 129.3 kN, respectively.

Slabs (SP9, SP10, and SP11) of Group III recorded a higher ultimate load compared to the second group “Group II” because of increased concrete thickness. The ultimate loads were 313.75 kN, 256.02 kN, and 212.10 kN for SP9, SP10, and SP11, respectively. 

Slab SP9 recorded an enhanced ultimate load with respect to all other slabs due to the concrete thickness and the small diameter Φ 8 of the GFRP reinforcement.

### 3.2. Ultimate Deflection

Table 5 shows the ultimate deflection for all slabs. For the control group, the ultimate deflation recorded was 6.75 mm and 4.89 mm for SP1 and SP2, respectively. This shows the effect of increasing the reinforcement ratio in decreasing deflection.

For Group I, Slab SP5 recorded the lowest deflection value of 3.72 mm compared to SP3 and SP4 and control slabs. The slabs SP3 and SP4 recorded a deflection of 2.47 mm and 4.91 mm, respectively.

For Group II, Slab SP6 recorded a higher deflection of 7.91 mm with an ultimate load-carrying capacity of 180.70 kN compared to Slabs SP7 and SP8, which recorded lower deflection values of 4.91 mm and 4.79 mm and an ultimate load of 149.30 kN and 129.30 kN, respectively, which agrees with Achillides and Pilakoutas [34].

**Figure 6:**
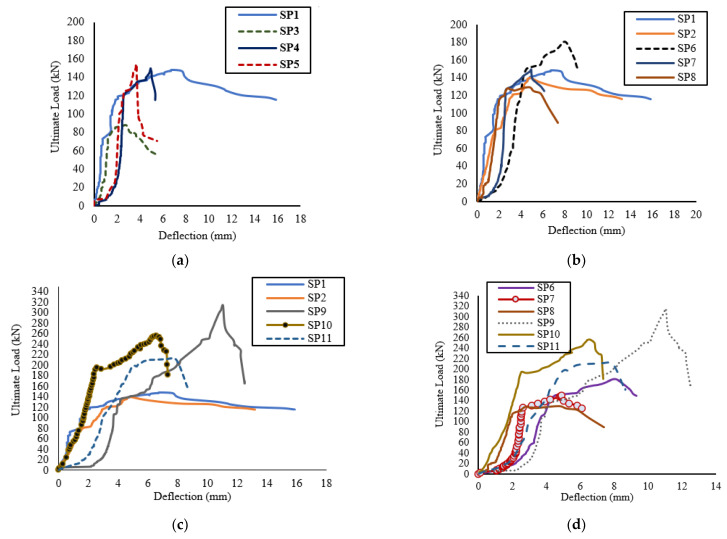
Load–deflection curves: (**a**) slabs with Φ 8 in control and Group I; (**b**) slabs with the same reinforcement ratio with different diameter (ts = 120 mm); (**c**) slabs with the same reinforcement ratio with different diameter (ts = 150 mm); (**d**) slabs with the same reinforcement ratio and different diameter (ts).

For Group III, Slab SP9 recorded the highest deflection of 11.03 mm with the highest ultimate load-carrying capacity of 313.75 kN compared to slabs of all groups. This indicated that the GFRP bars enhanced the loading-carrying capacity, deflections. and ductility when using small diameters, which increased the bond between concrete and bars, as shown in Figure 6 through the load–deflection curves for all slabs.

### 3.3. Crack Pattern and Mode of Failure

**Figure 7:**
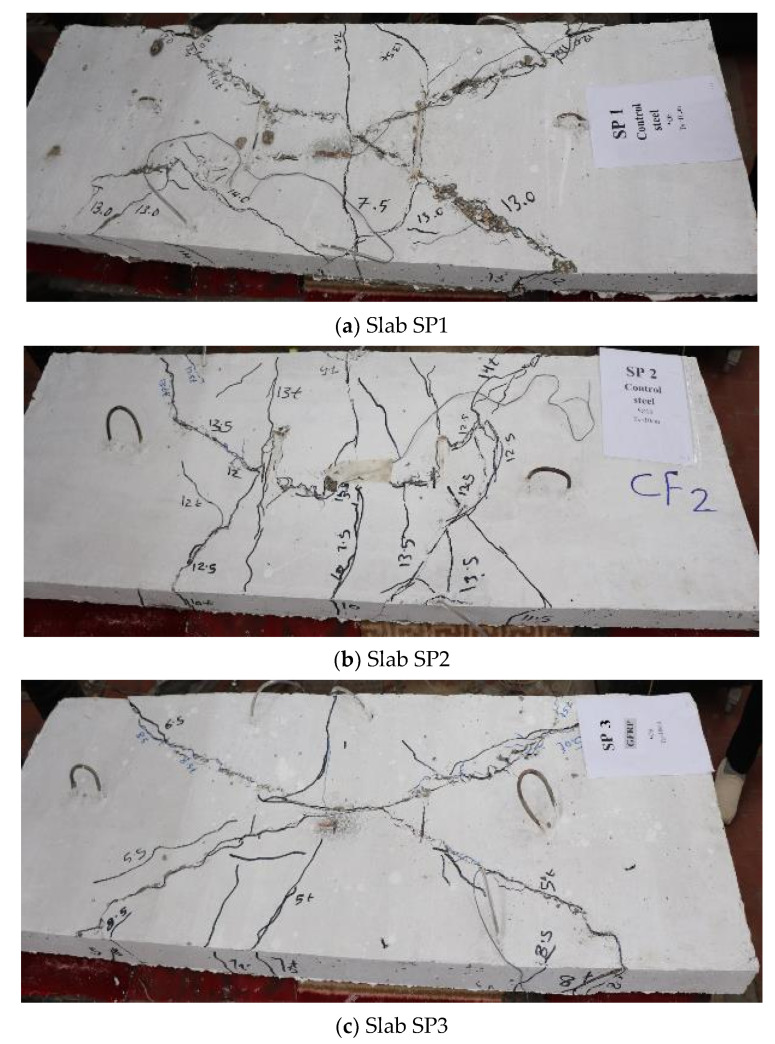

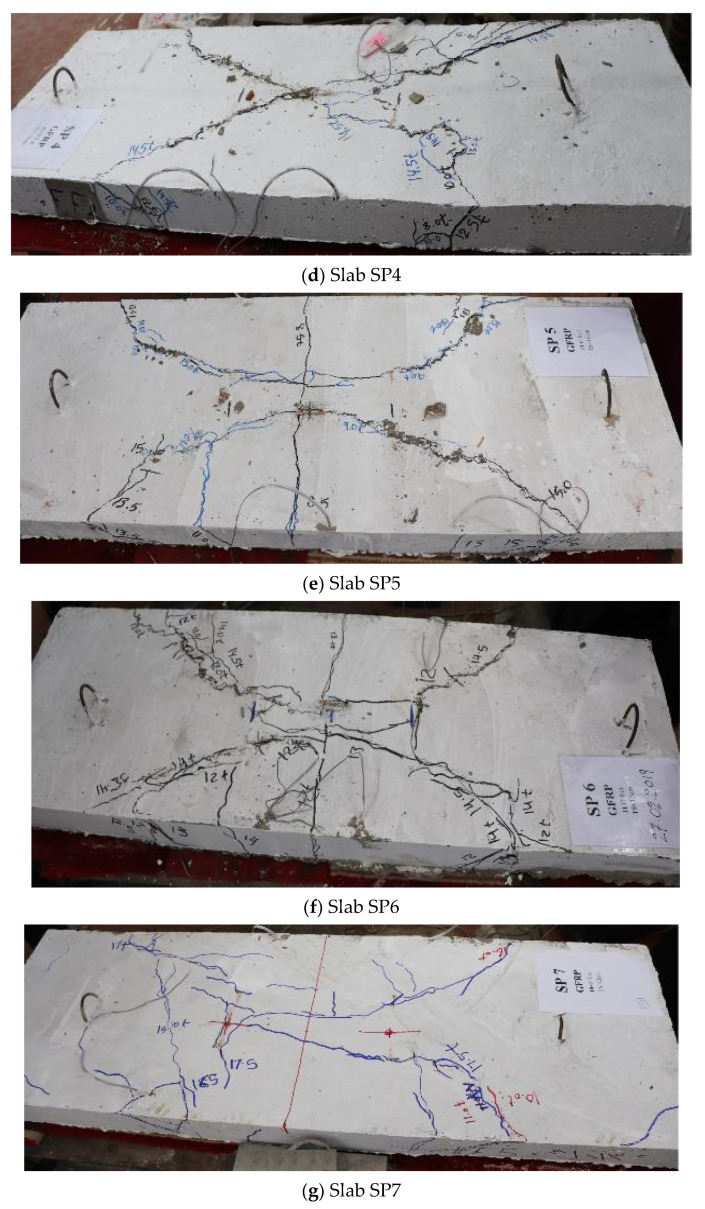

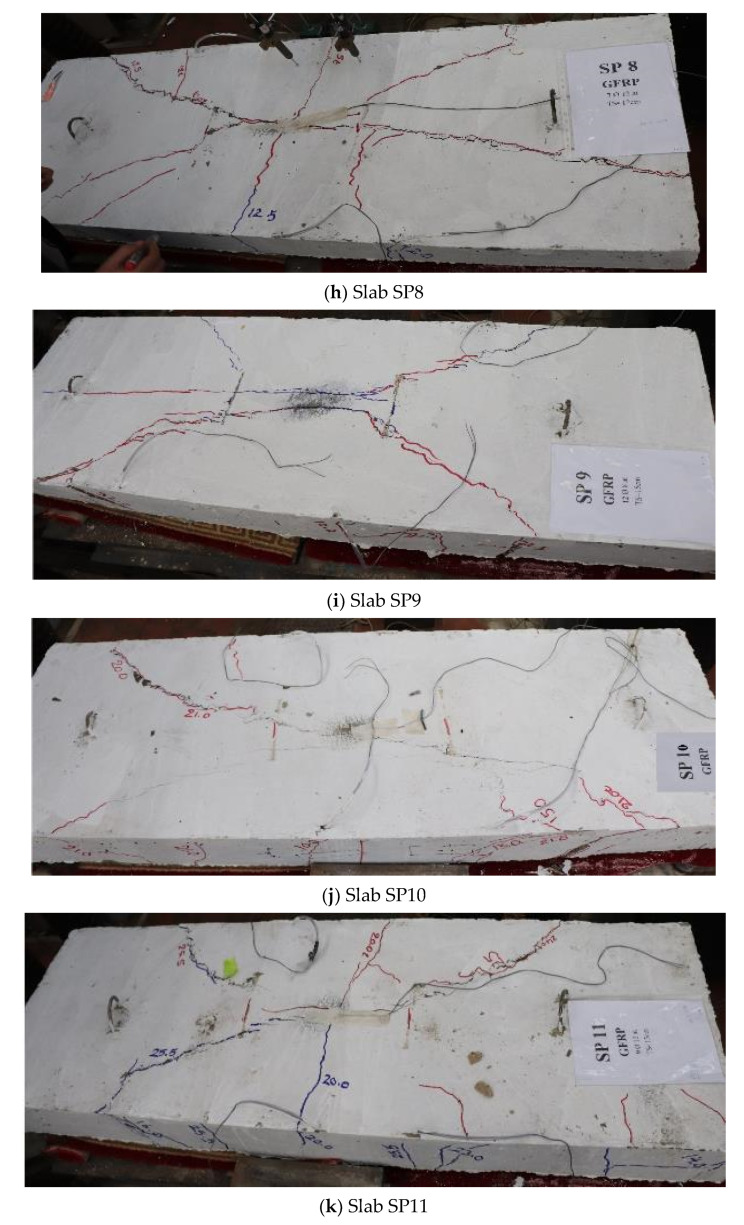
Crack pattern for all slabs.

**Figure 8:**
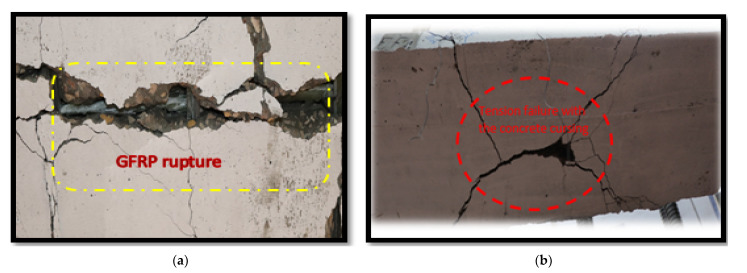
Crack pattern: (**a**) rupture of GFRP bars; (**b**) tension failure with concrete crushing.

Figure 7 shows the crack propagation for all slabs. Additionally, Table 5 shows the mode of failure for all slabs. Crack pattern for the control slabs SP1 and SP2 was propagated in the tension zone, as shown in Figure 7a, and the mode of failure was tension failure (TF). The behavior of Slabs SP3, SP6, and SP9 was the same. Although the concrete capacity was still able to carry load, the GFRP bars could not, so rupture failure (RF) occurred in the GFRP bars. For Slabs SP4, SP7, and SP10, the concrete and bars failed, together with compression and rupture failure (CC and RF, respectively). However, for Slabs SP5, SP8, and SP11, a decrease in crack number and propagation was noticed, as shown in Figure 8, and the mode of failure occurred as tension cracks and GFRP rupture failure.

## 4. Nonlinear Finite Element Analysis (NLFEA)

**Figure 9:**
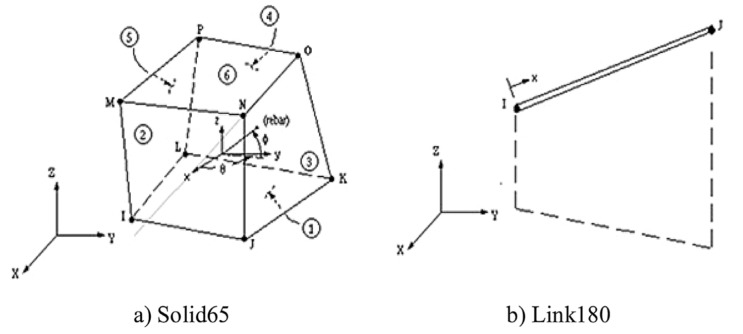
Element geometry: (**a**) Solid-65; (**b**) Link-180.

A finite element model was created to validate the experimental study using the ANSYS 2019-R1 [35] program. The Solid-65 element was employed for the representation of concrete, and the LINK-180 element was employed for steel and GFRP bar representation. Figure 9 indicates the Solid-65 and LINK-180 elements’ geometry.

### 4.1. Modeling

**Figure 10:**
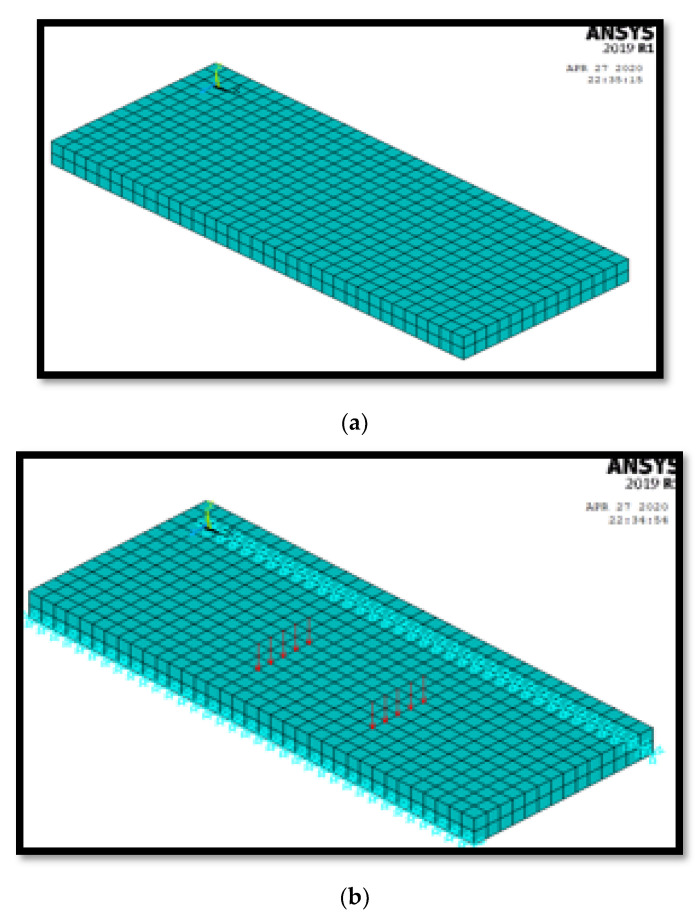
NLFEA model: (**a**) slab modeling; (**b**) restraints and applied loads.

The NLFE model was used to investigate the structural performance of HSC slabs reinforced with GFRP bars using ANSYS2019-R1 software, as indicated in Figure 10. in terms of ultimate load, ultimate deflection, and crack pattern for the modeled slabs.

### 4.2. NLFE Ultimate Load

Table 6 shows the ultimate loads obtained from NLFEA. For the control slabs SP1 and SP2, the ultimate load was 133.30 kN and 118.40 kN, respectively. For Slabs SP3, SP4, and SP5, the ultimate load was 76.42 kN, 119.40 kN, and 138.92 kN, respectively. For Slabs SP6, SP7, and SP8 the ultimate load was 153.60 kN, 134.40 kN, and 112.40 kN, respectively. For Slabs SP9, SP10, and SP11, the ultimate load was 247.86 kN, 215.05 kN, and 193.10 kN, respectively.

The enhancement in ultimate load for Slab SP9 compared with the control slabs led to concrete compressive strength and concrete thickness. The enhanced ratio is slightly low due to the small values of strain and the Young’s modulus of GFRP bars.

### 4.3. NLFE Deflection

**Figure 11:**
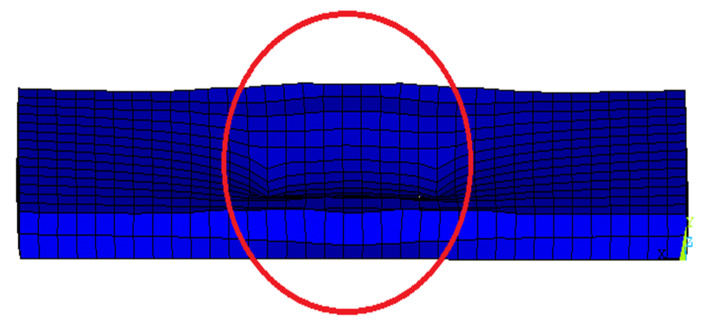
Sample of NLFEA deflection.

The NLFE deflections obtained are indicated in Table 6. Generally, the recorded deflection improved due to the use of GFRP bars with respect to control slabs. The deflection of SP1 was 6.10 mm at failure load, but it recorded an enhancement that varied between 60.0% and 45.0% for SP3, SP4, and SP5. For the second group, the deflections recorded were 6.72 mm, 4.41 mm, and 4.16 mm for SP6, SP7, and SP8, respectively. The enhancement was apparent in Slab SP8, which had the least compressive load with a ratio of 1.2 µ_fb_. This indicates the behavior of GFRP bars in enhancing the deflections, as shown in Figure 11.

### 4.4. Crack Pattern and Mode of Failure

**Figure 12:**
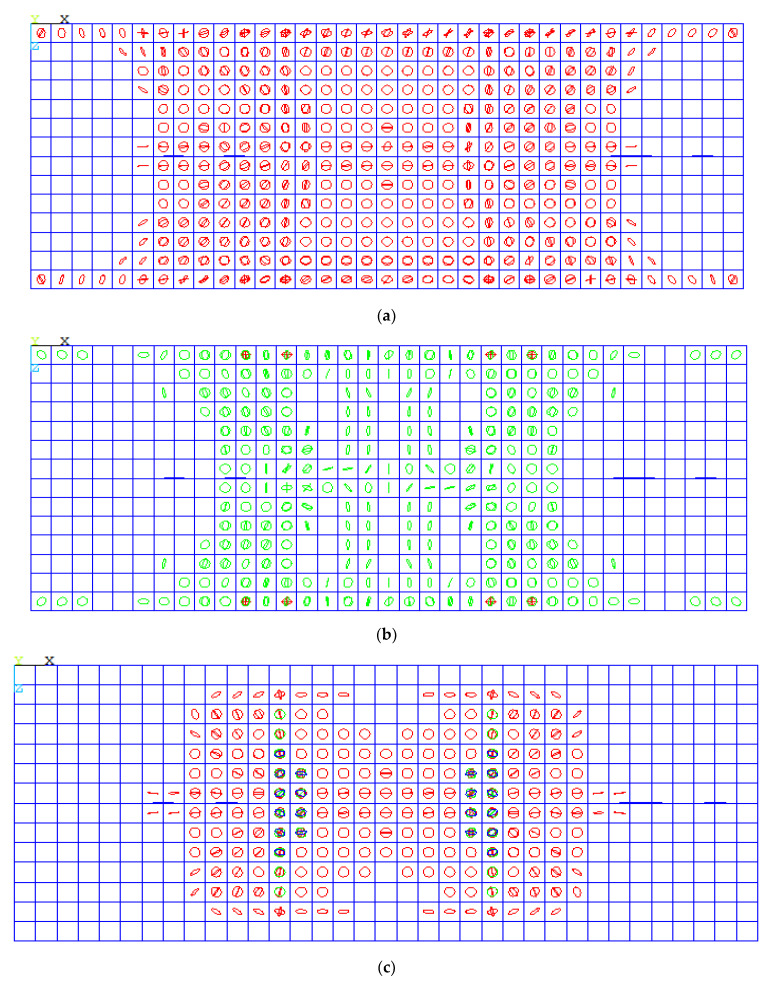

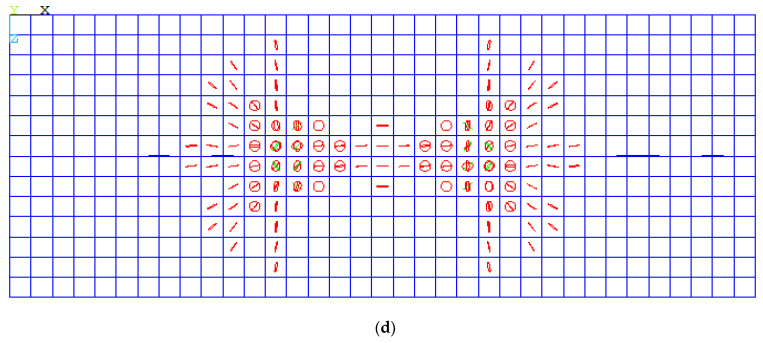
NLFE crack pattern: (**a**) control slabs; (**b**) Group I slabs; (**c**) Group II slabs; (**d**) Group III slabs.

The crack pattern of the control group featured crack propagation in the tension zone, as shown in Figure 12a. Additionally, the mode of failure was tension failure (TF) due to reinforcement failure. The behavior of SP3 and SP6 was the same, while the reinforcement was less than 0.8 µ_b_. So, the concrete capacity was still able to carry load, but the GFRP bars could not. Rupture occurred in GFRP bars, which was sudden rupture due to the brittle nature of GFRP bars, so there was RF in the bars. The mode of failure for the first group is the same for the second group in crack propagation and mode of failure. For slabs that had a reinforcement ratio of 1.2 µ_fb_, the failure was a combination of concrete cracks in the compression zone and rupture in the GFRP bars, as shown in Figure 12d. 

## 5. Comparisons between Experimental and NLFEA Results

There was good agreement between the experimental and ANSYS results. Comparisons were made between ultimate load, deflection, the first crack load, and crack pattern.

### 5.1. Comparison between Experimental and NLFE Ultimate Loads

**Figure 13:**
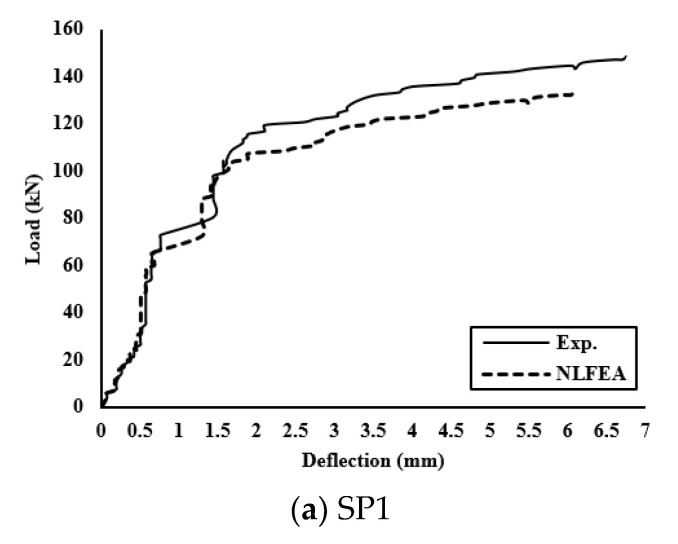

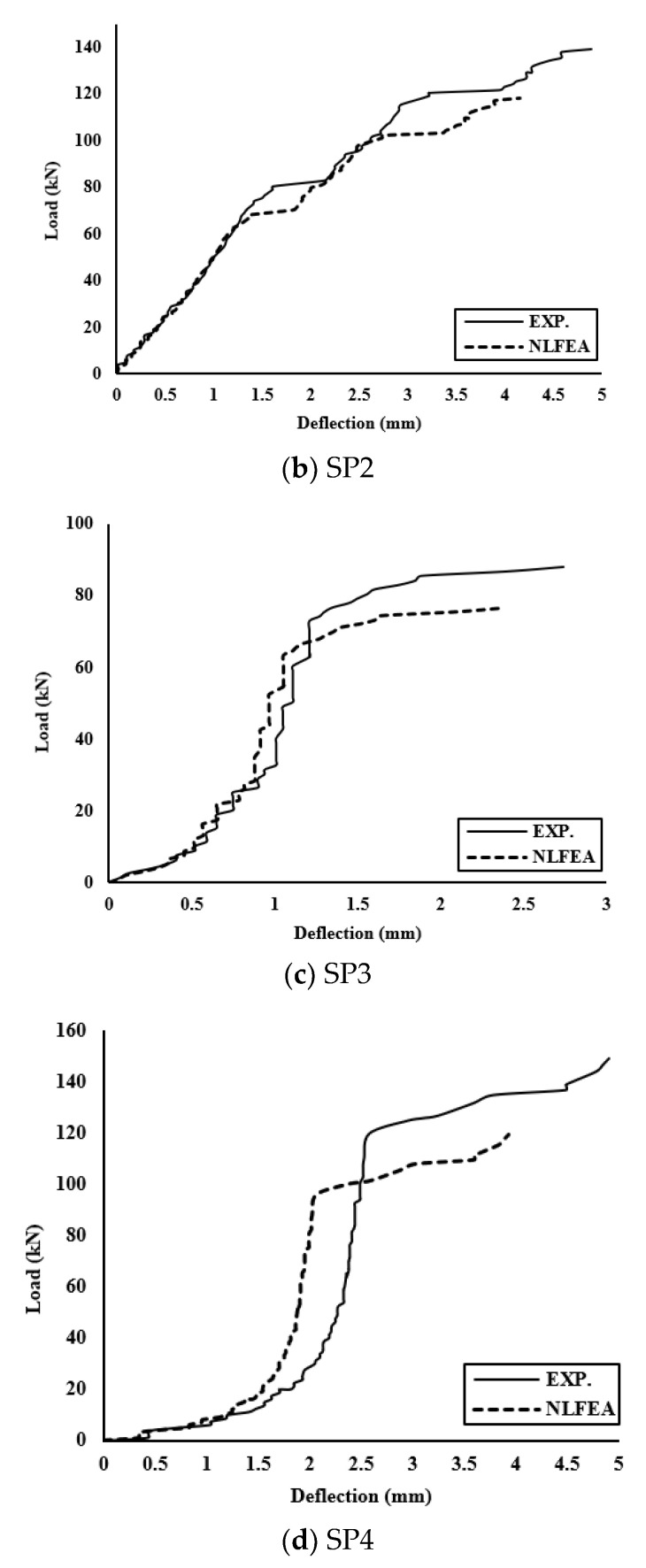

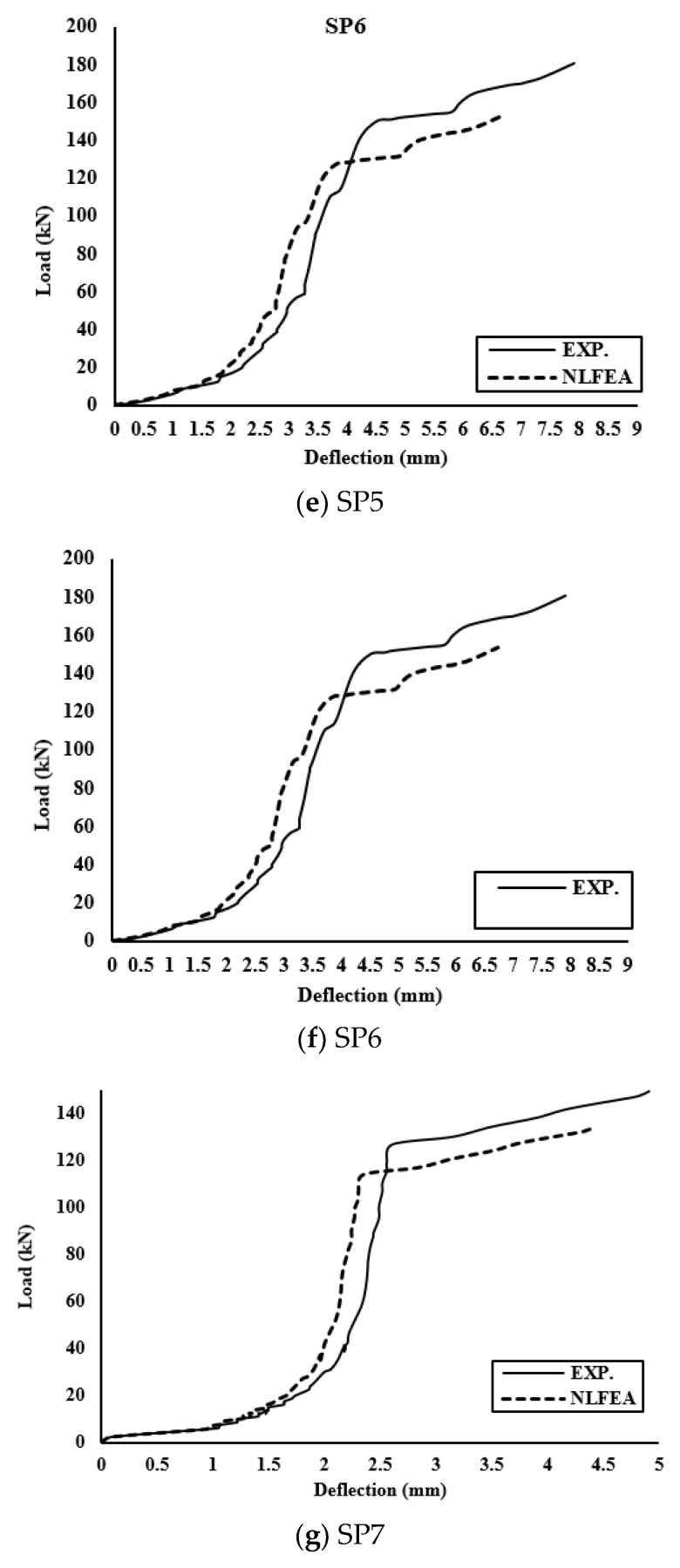

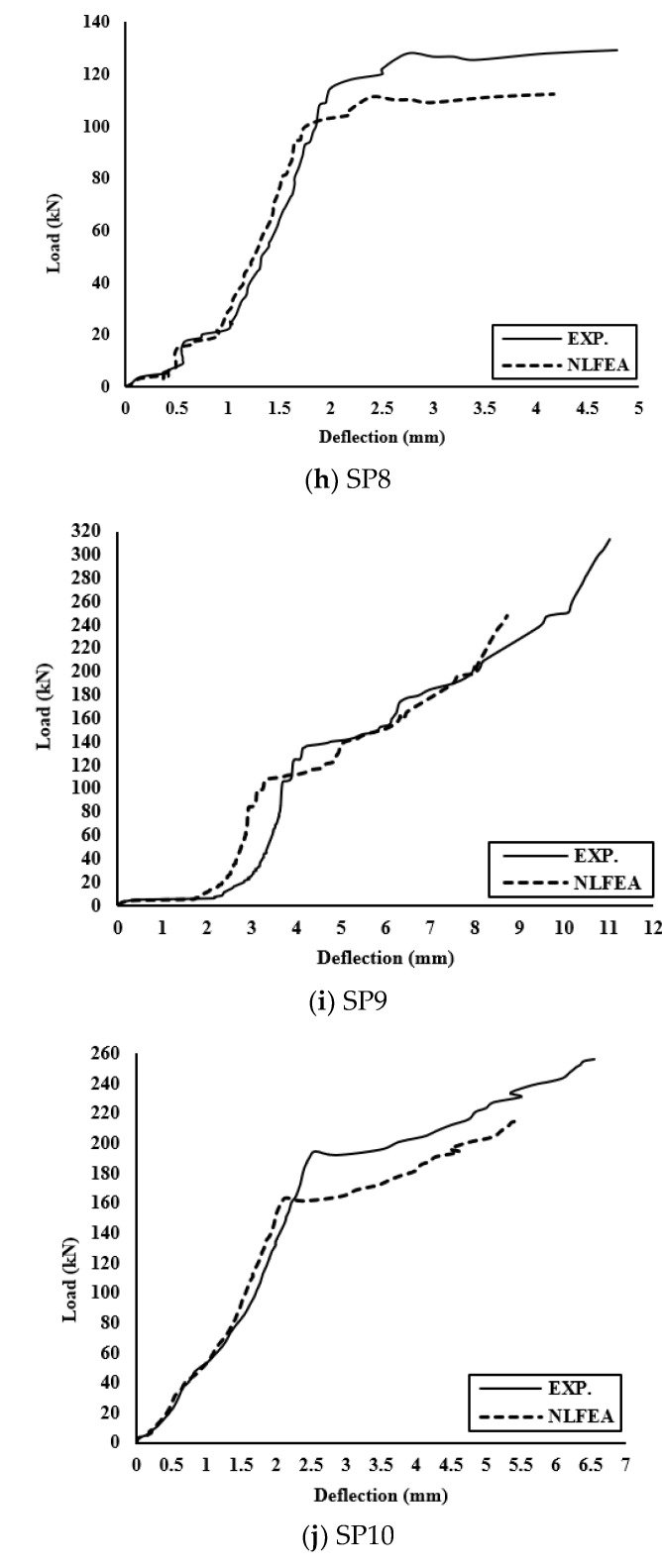

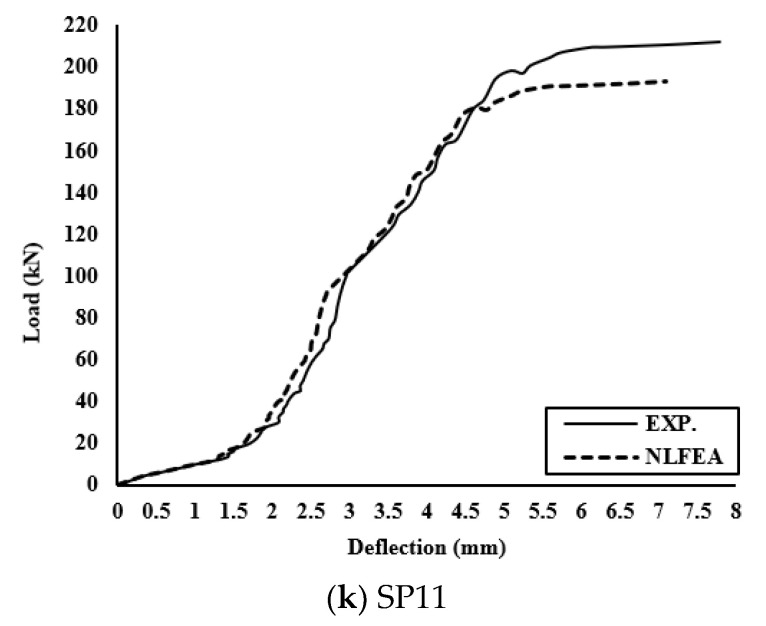
Comparisons between experimental and NLFE load–deflection curves for all slabs.

**Figure 14:**
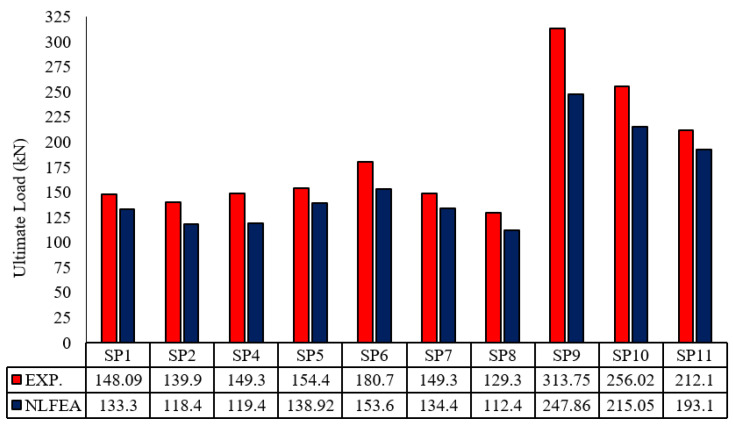
Comparisons between experimental and NLFE ultimate load.

Figure 13 shows good agreement between the experimental and analytical load–deflection curves. Comparisons between the obtained results for the different groups are shown in Table 7. Pu NLFEA/Pu exp. had an average ratio of 0.86. Group II of concrete reinforced with GFRP of the same diameter but different reinforcement ratios for SP3, SP4, and SP5, respectively, has an average of 0.86. Finally, for Group II and Group III, the average ratio of agreement for all specimens is 0.87 and 0.84. The variance of 0.0015 and standard deviation of 0.04 show the effect of using NLFEA in predicting the behavior of the tested slabs, as shown in Table 7 and Figure 14.

### 5.2. Comparison between Experimental and NLFE Deflections

**Figure 15:**
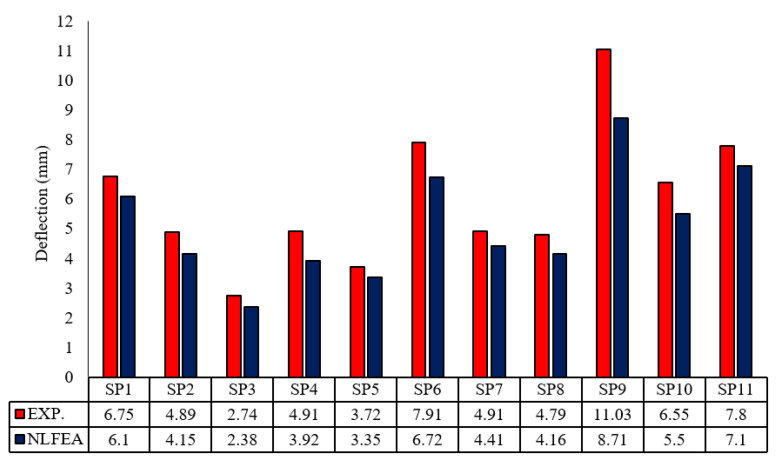
Comparisons between experimental and NLFE deflections.

Figure 15 shows the obtained deflections for all groups for both experimental and analytical studies. The load–deflection curves for the tested slabs and analytical results show good agreement, with an average of agreement of 86.0%. Table 7 shows a deflection ratio ∆_u NLFEA_/∆_u exp_. of the control group of 0.87, but for Group I, the ratios are 0.87, 0.79, and 0.90 for SP3, SP4, and SP5, respectively, and the average ratio of agreement is 0.85. This indicates that the analytical models provided an acceptable load–deflection response, as shown in Table 7. For all groups, the average of ∆_u NLFEA_/∆_u exp_ is equal to 0.86, with a coefficient of variance and standard deviations of 0.0016 and 0.041, respectively.

### 5.3. Comparison between Experimental and NLFE Crack Patterns and Mode of Failure

**Figure 16:**
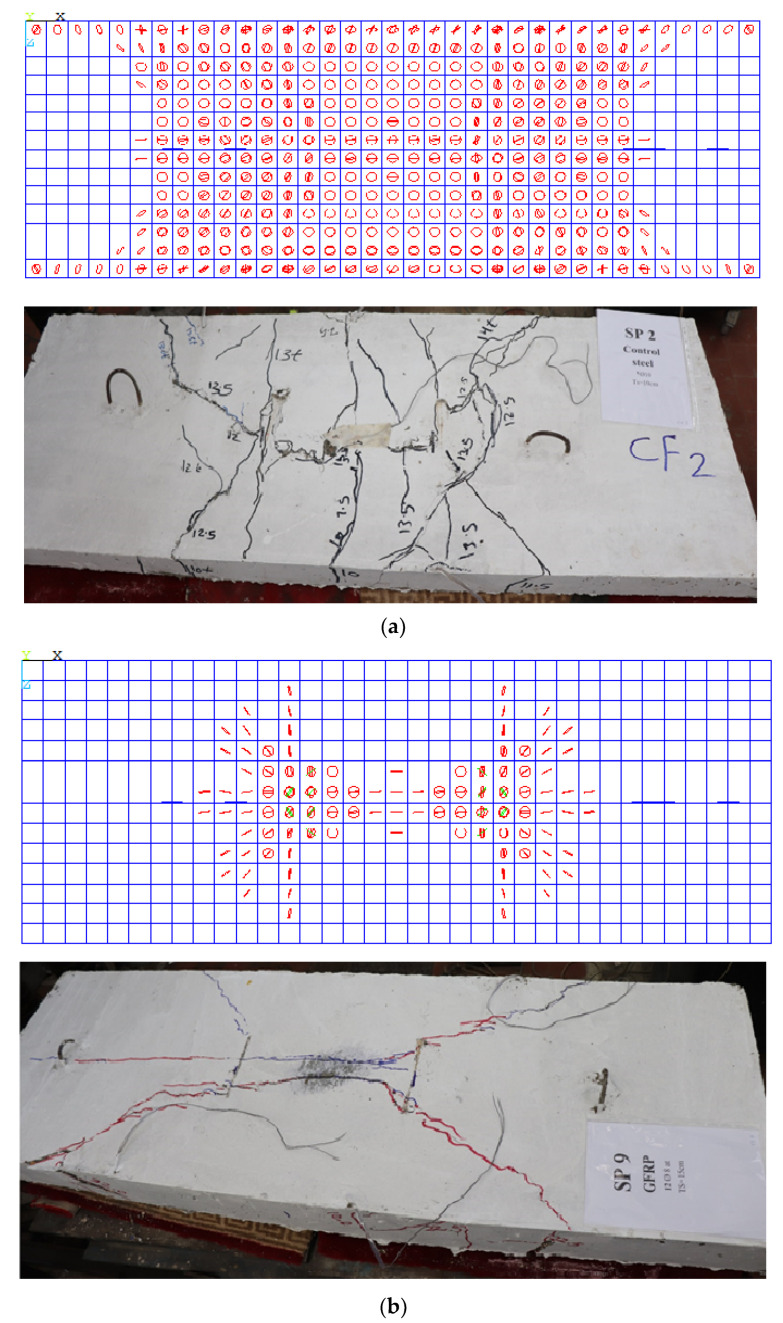
Comparisons between experimental and NLFE crack patterns: (**a**) slabs reinforced using steel bars; (**b**) slabs reinforced using GFRP.

The crack pattern for the control slab with a steel reinforcement started with crack propagation in the tension zone for the experimental and analytical slabs, as shown in Figure 16a, showing tension failure (TF).

However, for Slabs SP5 to SP11 reinforced with the same reinforcement ratio, a higher ultimate load, lower deflection, and decreased cracks were obtained, showing tension cracks with low propagation, as obtained from the experimental patterns. The crack patterns show good agreement between the NLFEA and experimental results.

## 6. Conclusions

Based on the experimental and the analytical studies, the following conclusions can be drawn:

1. Using reinforcement areas of the GFRP bars less than or equal to µ_b_ led to brittle failure in GFRP bars and concrete crushing with rupture GFRP bars, respectively.
2. The behavior of the tested GFRP-reinforced slabs was bilinear elastic until failure.
3. There was an enhancement in deflections and crack patterns for slabs reinforced using GFRP bars, especially for equal reinforcement areas.
4. The NLFEA obtained an acceptable agreement with the experimental study in terms of the ultimate loads, ultimate deflection, and crack pattern.
5. The agreement between the experimental and analytical study was approximately 86.0% with a standard deviation of 0.04 and a coefficient of variance of 0.0015.

## Figures and Tables

**Table 1:** Material weights.

Materials	*Per m*^3^*of Concrete* (f_cu_ = 60 MPa)
Cement	575 kg/m^3^
Coarse aggregate	1100 kg/m^3^
Fine aggregate	580 kg/m^3^
Water	138 kg/m^3^
Silica fume	50 kg/m^3^
Superplasticizer	18 kg/m^3^

**Table 2:** Compressive strength test results.

Cubes	Compressive Strength (MPa)
	28 Days
C−1	63.9
C−2	68.2
C−3	66.7
Average	66.3

**Table 3:** GFRP bar tensile stresses.

Diameter (mm)	Tensile Strength (MPa)
8	490
10	650
12	750

**Table 4:** Specimen details.

Specimen Group	Specimen ID	Thickness (mm)	Diameter (mm)	Reinforcement Ratio %	RFT. Type
Control	SP1	100	⌀ 8	0.16 µb	Steel
	SP2	100	⌀ 10	0.24 µb	Steel
Group I	SP3	100	⌀ 8	0.80 µfb	GFRP
	SP4	100	⌀ 8	1.00 µfb	GFRP
	SP5	100	⌀ 8	1.20 µfb	GFRP
Group II	SP6	120	⌀ 8	1.20 µfb	GFRP
	SP7	120	⌀ 10	1.20 µfb	GFRP
	SP8	120	⌀ 12	1.20 µfb	GFRP
Group III	SP9	150	⌀ 8	1.20 µfb	GFRP
	SP10	150	⌀ 10	1.20 µfb	GFRP
	SP11	150	⌀ 12	1.20 µfb	GFRP

**Table 5:** Experimental results.

Specimen Group	Specimen ID	First Crack (kN)	Ultimate Load (kN)	Ultimate Deflection Δ_u_ (mm)	Mode of Failure
Control	SP1	75	148.00	6.75	FF
	SP2	75	139.00	4.89	FF
Group I	SP3	50	87.85	2.74	GR
	SP4	80	149.30	4.91	GR + TF
	SP5	85	154.40	3.72	CCT
Group II	SP6	100	180.70	7.91	CCT
	SP7	120	149.30	4.91	GR
	SP8	125	129.30	4.79	GR
Group III	SP9	100	313.75	11.03	GR
	SP10	150	256.02	6.55	CCT
	SP11	200	212.10	7.80	CCT

Concrete cracking and tension (CCT) cracks, GFRP rupture (GR), flexural failure (FF).

**Table 6:** NLFEA results.

Specimen Group	Specimen ID	First Crack (kN)	Ultimate Load (kN)	Δ_NLFA_ (mm)
Control	SP1	50	133.30	6.10
	SP2	50	118.40	4.15
Group I	SP3	50	76.42	2.38
	SP4	50	119.40	3.92
	SP5	50	138.92	3.35
Group II	SP6	70	153.60	6.72
	SP7	70	134.40	4.41
	SP8	70	112.40	4.16
Group III	SP9	82	247.86	8.71
	SP10	82	215.05	5.50
	SP11	82	193.10	7.10

**Table 7:** Comparisons between experimental and NLFEA results.

Specimen Group	Spec. ID	Experimental Load (kN)		Analytical Load (kN)		Δ (mm)		$\frac{\mathrm{Pu}\;\left(\mathrm{NLFE}\right)}{\mathrm{Pu}\;\left(\exp\right)}$		$\frac{\Delta \;\left(\mathrm{NLFE}\right)}{\Delta \;\left(\exp\right)}$
		First Crack	Ult. Load	First Crack	Ult. Load	Δ_exp_	Δ_NLFE_	First Crack	Ult. Load	
Control	SP1	75	148.00	50	133.30	6.75	6.10	0.67	0.90	0.90
	SP2	75	139.00	50	118.40	4.89	4.15	0.67	0.85	0.84
Group I	SP3	50	87.85	50	76.42	2.74	2.38	1.0	0.87	0.87
	SP4	80	149.30	50	119.40	4.91	3.92	0.62	0.80	0.79
	SP5	85	154.40	50	138.92	3.72	3.35	0.59	0.90	0.90
Group II	SP6	100	180.70	70	153.60	7.91	6.72	0.70	0.85	0.85
	SP7	120	149.30	70	134.40	4.91	4.41	0.58	0.90	0.89
	SP8	125	129.30	70	112.40	4.79	4.16	0.56	0.87	0.87
Group III	SP9	100	313.75	82	247.86	11.03	8.71	0.82	0.79	0.79
	SP10	150	256.02	82	215.05	6.55	5.50	0.55	0.83	0.84
	SP11	200	212.10	82	193.10	7.80	7.10	0.41	0.91	0.91
Average								0.65	0.86	0.86
Variance								0.019	0.0015	0.0016
Standard Deviation								0.15	0.04	0.041

## Data Availability

All data included in this study are available upon request by contact with the corresponding author.
